# Implementation and Sustainability of a Pharmacy-Led, Hospital-Wide Bedside Medication Delivery Program: A Qualitative Process Evaluation Using RE-AIM

**DOI:** 10.3389/fpubh.2019.00419

**Published:** 2020-01-22

**Authors:** Beth Prusaczyk, Amanda S. Mixon, Sunil Kripalani

**Affiliations:** ^1^Department of Medicine, Washington University School of Medicine, St. Louis, MO, United States; ^2^Department of Medicine, Vanderbilt University Medical Center, Nashville, TN, United States

**Keywords:** implementation, hospital, sustainability, RE-AIM, qualitative

## Abstract

**Background:** Few studies of hospital-based implementation assess sustainability or collect formal implementation outcomes, in part because the emphasis is often on initial adoption and rapid cycles of improvement. The purpose of this process evaluation was to assess the implementation of a pharmacy-led, hospital-wide program and contribute to the literature by collecting formal implementation outcomes, including sustainability.

**Methods:** This was a qualitative process evaluation of a program that delivers discharge medications and related education to hospitalized patients' bedside prior to discharge. Semi-structured interviews were conducted with the program's key stakeholders to assess the program's implementation barriers and facilitators as well as its potential for sustainability. An interview guide was created based on the RE-AIM constructs of Reach, Adoption, Implementation, and Maintenance. Effectiveness was not assessed due to an ongoing effectiveness evaluation by another team. Each interview was coded by two independent coders and any discrepancy was adjudicated by a third, independent coder.

**Results:** Twelve stakeholders were approached and all agreed to be interviewed. Related to providers' decisions to *adopt* the program, key themes emerged around the different priorities of nurses and physicians, which has implications for how program leadership promoted the program to these different stakeholder groups. Key *implementation* barriers included the nature of hospital provider rotations and turnover, which led to confusion on who could use the program and to whom providers should direct program-related questions. Key *implementation* facilitators included the enthusiasm of program staff and identified champions on the units. Themes related to *maintenance* or sustainability included the need to continually generate buy-in and educate providers about the program and allowing program staff and leadership to remain nimble and adapt their operations to meet evolving needs.

**Conclusions:** The results suggest that in an environment in which rapidly achieving improvement is often the focus more than maintaining that improvement, strategies to achieve successful implementation may not be sufficient to achieve successful sustainment. New strategies are likely needed to address the unique barriers to sustaining a program once initial adoption and implementation is complete.

## Introduction

Bedside medication delivery programs, commonly referred to as “meds to beds” or “meds in hand,” involves delivering discharge medications to hospitalized patients' bedside prior to discharge and often includes a medication education component (1, 2). This intervention, or program, can be provided by hospital pharmacies, on-site affiliated outpatient pharmacies, or third-party retail pharmacies. In this study, the specific program was conceptualized and initiated by the on-site hospital-affiliated outpatient pharmacy as a quality improvement initiative for the purpose of improving patients' transition from hospital to home.

These programs have been shown to significantly reduce 30-day hospital readmissions (in one study the reduction was greater among older adult patients) (1) and emergency department visits (2). Although not empirically tested, hospitals also report that these programs increase the number of patients actually obtaining their discharge medications by removing common barriers related to payment and transportation, increase patient satisfaction, and reduce costs (3, 4).

To our knowledge, there has been no study of the implementation of these programs. A recent systematic review of the barriers and facilitators to the implementation of hospital-based interventions by Geerligs and colleagues found that the barriers and facilitators fell into three domains: system-, staff-, and intervention-level (5). System-level barriers and facilitators included the physical structure/environment, resources, culture, and external pressures such as reporting guidelines and regulations. At the staff level they included awareness, attitudes, commitment, role identity, skills, ability, and confidence. Intervention-level barriers and facilitators included the ease of integration, strength of the evidence, and available support. The authors found that there was considerable interaction between the domains as well (5). For example, a response to an intervention-level barrier (lack of flexibility) might influence a staff-level facilitator (confidence in ability to deliver the intervention with fidelity) which in turn might affect the system (a change in culture).

However, the authors also noted a number of areas in which the included studies fell short. First, few studies addressed sustainability thus it was unclear how the barriers and facilitators to initial implementation impacted long-term sustainability (5). Similarly, a majority of studies included in the review assessed implementation only anecdotally and did not collect formal implementation outcome data, making it hard to generalize across different studies (5).

The current study addresses these limitations by evaluating the implementation of a pharmacy-led, hospital-wide intervention 3 years after implementation and using RE-AIM (6) to guide the collection of implementation outcome data, including sustainability. Definitions of sustainability, or maintenance, vary, and include constructs such as those related to the passage of time, funding support, or the presence of workplace policies on the intervention. For the purpose of this study, we used the passage of time as our criterion for sustainability and, consistent with the definition of Maintenance from the RE-AIM framework, we assessed sustainability at least 6 months after initial implementation of the intervention (6).

## Methods

### Setting

This study was conducted at Vanderbilt University Medical Center. Vanderbilt University Hospital (VUH) has 834 beds, 36 nursing units, and provides a variety of services including medical, surgical, and specialty care. The hospital-affiliated, on-site outpatient pharmacy serves the adult hospital for discharge prescriptions, as well as all outpatient clinics at the medical center.

### Intervention

The Meds to Beds program, launched in March of 2016, fills the patients' discharge medications and delivers them to their hospital bedside, where education about their medications is also provided. Through the program, the pharmacy processes patients' insurance (just as a third-party pharmacy would) and, if the patient cannot pay for their medications, utilizes appropriate discount or charity programs to assist patients in covering the costs. Ideally, the inpatient providers (physicians or nurses) send patients' discharge medication prescriptions to the pharmacy as soon as those orders are written, and the pharmacy processes those orders and delivers the medications to the patients' bedsides as soon as possible. On average, it takes the pharmacy approximately 2 h from the time they receive the orders to process them and deliver the medications. However, as we will discuss below, this ideal process is not always feasible.

The program was initially provided to only a few units on a few services (e.g., a single unit on surgery, a single unit on medicine) but within 6 months expanded to the entire hospital. The program has designated full-time staff including pharmacy technicians and pharmacists. In the beginning pharmacists delivered the medications and provided the education, but as the program expanded the increased demand caused program leadership to adapt the program. With this adaptation, pharmacy technicians now deliver the medications and use a tablet computer to facilitate a two-way video call between the patient in the hospital room and a program pharmacist in the pharmacy to provide education. The program is “opt-out,” where patients are able to choose not to use the program or the hospital pharmacy if they prefer to use an outside pharmacy.

### Design

We used the RE-AIM framework to guide our evaluation. RE-AIM stands for Reach, Effectiveness or Efficacy, Adoption, Implementation, and Maintenance (6) RE-AIM was the appropriate framework because of its applicability to an evaluation effort and its specificity of constructs. This specificity also addresses the gap found in Geerligs' review that studies of hospital-based interventions failed to collect generalizable implementation outcomes.

We conducted brief, semi-structured interviews with various program stakeholders and hospital providers. We structured our interview guide around Reach, Adoption, Implementation, and Maintenance. A separate effectiveness study was being conducted at the same time as this implementation evaluation; therefore, we chose not to measure program effectiveness in the current study.

### Participants

We worked with program leadership to identify key stakeholders in the implementation of the program to approach for interviews. These included pharmacy and nursing leadership, program staff, physicians, and nurses. We also utilized snowball sampling techniques, asking interviewees for the names and information of others they thought we should speak to. All identified stakeholders were employees of Vanderbilt University Medical Center. This project was reviewed by the Institutional Review Board at Vanderbilt University Medical Center and was deemed a quality improvement project evaluation and not human subjects research thus informed consent was not obtained from the providers.

### Data Collection

The program was adopted (i.e., launched) in March 2016 and was fully implemented hospital-wide by October 2016. Data collection for this evaluation took place over a 5-month period from November 2017 to March 2018, beginning approximately 21 months after adoption and 13 months after implementation was complete. Semi-structured interviews were conducted by a single interviewer (author BP). A structured interview guide based on the RE-AIM framework was used and is available (see Supplementary Material File 1). For the purposes of this study, the operationalization of the RE-AIM constructs were adapted and these adaptations can be seen in the codebook (see Supplementary Material file 2). Specific questions about how the program was “pitched” to the interviewee, the barriers and facilitators to implementation, any adaptations made to the program since it began, and factors impacting the program's sustainability were included. The interviews were conducted in-person and lasted approximately 30 min. Because these interviews often occurred in patient-care settings they were not audio recorded for privacy reasons. However, the interviewer took detailed and extensive notes during and immediately after the interview.

### Data Coding and Analysis

A codebook was created based on RE-AIM. This codebook was created by author BP and sent to authors ASM and SK for input. Clarifications and additional detail was added to the codebook based on their input. The purpose of this codebook was to define the constructs of the RE-AIM framework and to guide coders in assigning text to one of the four constructs (Reach, Adoption, Implementation, Maintenance).

BP and AM then independently coded each interview to identify blocks of text that represented one of the four constructs. The results of this first round were reviewed and any discrepancy was adjudicated by SK. Then BP and AM conducted a more detailed second round of coding to identify specific themes within these blocks of text. The results of this second round were discussed by BP, AM, and SK, and consensus was reached on the emergent themes.

## Results

A total of 12 interviews were conducted. The two program leaders who assisted in identifying stakeholders were interviewed in addition to one representative from pharmacy operational leadership, one representative from nursing operational leadership, one pharmacy supervisor, one program supervisor, one program pharmacist, one inpatient unit pharmacist (not affiliated with the program), two attending physicians (one of whom was a champion of the program when it began), and two nurses. These nurses were specifically “patient flow nurses” which is a type of nurse at the hospital tasked with facilitating timely and quality discharges.

### Reach

A key facilitator to the *reach* of the program was the program being “opt-out,” meaning only when patients expressed wishes to not use the program did they actually not receive it.

“*The opt-out model is extremely important. The patient is sick, the caregiver is in the room tapping their foot, and everyone just wants to go home.” – Pharmacy Administrator*

In turn, stakeholders noted that the main reason patients declined to use the program was when they had a strong, personal relationship with their hometown pharmacy.

“*If they want to go to their local pharmacy that's fine but if the default is they get the medications here, at least they have it in their hand when they leave and they don't ultimately come back.” – Pharmacy Administrator*

“*Patients who have a local, independent pharmacy are “ride or die” with their pharmacy because the pharmacist there knows their history and knows their families so they will often decline (the program).”– Inpatient Unit Pharmacist (not affiliated with the program)*

### Adoption

A common theme discussed related to providers decision to adopt the program was that stakeholders have not just different priorities but competing priorities. This was apparent when interviewees discussed how the program was “pitched” to them or how they pitched it to others. Depending on what the actual or perceived priorities were of a stakeholder group, the pitch changed and in some cases these priorities were mutually exclusive. For example, priorities for attending physicians were related to the comprehensiveness of the program and the services—medication education and assistance with payment—it provides to their patients.

“*It was described that patients would get pharmacy education around their medications and previously I think it was an assumption on the nursing staff part that physicians did counseling and the physicians thought the nurse or pharmacy did the counseling so it didn't happen. With Meds to Beds it was actually guaranteed that the pharmacist would do it.” – Attending 1*

These additional services may be priorities for physicians because they recognize their potential effect on reducing readmissions, which was also stated as a priority for physicians.

“*For physicians it is all about readmissions and why readmissions occur—patients not getting prescriptions filled, reasons why they don't get prescriptions filled. And how we help with those things.” – Program Pharmacy Supervisor*

However, the provision of these additional services takes additional time, which was in conflict with the priorities of nurses. A key quality metric on which nursing units are monitored is what time patients actually leave the hospital.

“*[I tell the nurses] it will help decrease your work[load] and make your discharges quicker.” – Patient Flow Nurse 1, describing how she sells the program to other nurses*

Likewise, if nurses prioritize expedient discharges but patients leave too quickly without receiving appropriate arrangements and counseling, this could conflict with best practices for reducing readmissions.

### Implementation

Another instance where competing priorities related to the timing of discharge presented was during the implementation of the program. One of the most frequently cited barriers to implementation was the amount of time it took to deliver the medications. Physicians and nurses reported that the medications were often delivered later than they would prefer which delayed discharge.

“*I think some people didn't buy into it because they thought it would delay discharge. What do we do if their [the patient] ride is there but they don't have their meds? Do we reprint the prescriptions and let them go? Or spend extra time to call pharmacy and see when the meds will be there? Even now I still think about timing.” – Attending*

The program staff and leadership were made aware of this complaint from providers early during implementation and attempted to address the barrier by requesting that providers order their discharge medications as early as possible. The program often received patients' medication orders in the morning (usually mid-morning after team rounds) for a same-day, afternoon discharge. The pharmacy, on average, takes 2 h after receiving the prescription orders to process and deliver the medications. In order to get the medications delivered sooner, the program asked that providers send the orders to the pharmacy as soon as possible.

“*If there's a way to just start sending prescriptions down sooner that would be helpful. Sometimes they send them down the day before but it's at the end of the day at 6:29 p.m. And I mean, did you just now decide that patient was going home tomorrow? So if they could send the prescriptions down in real time that would help.” – Program Supervisor*

However, providers expressed concern about sending the orders sooner.

“*There was a fear in the beginning that if you sent the prescriptions down a day early, is the patient going to get them? Will they actually appear the next day?” – Attending 1*

“*And this will just be impossible on different services. On a teaching services the intern isn't going to prescribe until they've rounded with the attending. It's just not going to happen. So that won't ever happen early.” – Attending 2*

This tension—providers wanting the medications sooner but not always being able to follow the proposed solution of sending the orders sooner—is another example of not just different stakeholder' priorities but of conflicting priorities in which no “silver-bullet” solution exists.

Another recurring theme across the interviews was the challenge of implementing a program in an environment with complicated care structures and frequent planned and unplanned staff changes. VUH does not have geographic localization for most medical services, where physicians on a given rotation see all of their patients in the same hospital unit or location (exceptions include contained units like the Intensive Care Unit or Geriatrics). Instead, the hospital has a structure where physicians admit patients across different units and floors. The nursing staff is unit-based, however.

In addition to this unique structure, attendings, residents, and interns commonly rotate on and off teaching services at 2-week intervals. Attendings on non-teaching services rotate more frequently, usually every 5 days or so. Also, not unique to VUH is the typical, unanticipated staff turnover that all hospitals experience across all staff members.

The unique structure of VUH, the typical physician rotation schedule, and turnover of providers and staff were reported to have significant impact on the implementation of the program. First, program leaders and staff said the structure made the decision about where to begin rolling out the program difficult. If they decided to go with a physician-based rollout around specific medical or surgical teams, then it became challenging for the nurses, who were unit-based. Because the nurses on a single unit cared for the patients of numerous physicians, they had to remember which physicians were assigned the program and therefore which patients could receive the program. The alternative—a service- or unit-based rollout—would be challenging to physicians because they would have to remember which units were assigned the program and therefore which patients on their service they could enroll in the program based on what unit the patient was on.

“*Every unit has a different process for discharge. We went live on 7 North [service] because they had some familiarity with the Meds to Beds process then we went to the Riven [Hospitalist] Services. But Riven is everywhere with certain beds on certain units. So we had to pull back and rethink.” – Program Leader*

“*There was a big decision about which service to approach first. Do we go floor by floor? We started with one service spread across all these floors. Then communication and education with providers were really difficult.”– Program Pharmacy Supervisor*

The program leaders reported that they quickly learned their initial plan to start with one hospital service and slowly rollout the program sequentially to others was not feasible due to the reasons mentioned above (physicians had to remember which units and therefore which patients were eligible for the program).

“*They tried to roll out the program too quickly when they didn't have the staff. And then it was taking too long to get scripts and if the nurses have one bad experience or don't know who to contact they will write off the program as not useful. They don't have time to try things more than once.” – Patient Flow Nurse 1*

These challenges led the program to scale-up hospital-wide shortly after starting.

“*So we decided to roll out to everywhere and then go around and educate everyone. Because not being live on all floors was causing problems.” – Program Leadership*

It was this next phase of implementation—educating providers about the program—where providers' rotations and staff turnover caused challenges, according to multiple interviewees. Education about the program to providers included in-person instruction delivered by program staff to providers and reminder materials such as posters and flyers posted around the unit.

“*The hospital has high turnover, residents come through, and suddenly no one knows about us.” – Program pharmacist*

“*I would like to go to each unit and find the person who is going to—just someone who I can explain the program to and clear up any misconceptions. Because some people think they can't start the process until the patient is ready to discharge and I want them to know they can start it much earlier. But you know you have new residents coming in and out and they are hearing about the program from someone else and it's not the full story.”– Program Supervisor*

“*Residents need formal, in-depth education on the program from their attendings. If it's someone from the program delivering it, then it just seems like is a vendor wanting the residents to use their service.”– Patient Flow Nurse 1*

These comments highlight the challenges that presents to a program that is implemented hospital-wide.

Despite these significant barriers to implementation, interviewees also noted two major facilitators: patient flow nurses and the positive attitudes of the program staff.

Patient flow nurses are nurses hired for the specific purpose of facilitating patients' discharges, including ensuring the patients' hospitalizations and discharges are timely and high quality. While the hospital did not begin employing patient flow nurses because of the Meds-to-Beds program, their hiring did coincide with the program's rollout, with patient flow nurses starting only a few months after the program began. Multiple program staff commented on the importance of patient flow nurses in the program's implementation and future sustainability.

“*We got patient flow nurses about a year in and that made a huge difference. One of their chief goals is to get fast discharge. The hospital implementing them was a lucky break for the program. “– Program Pharmacy Supervisor*

“*The PFNs [Patient flow nurses] are great. They have our backs and they have similar barriers in their jobs. The perception is that Meds to Beds is taking too long but Pharmacy doesn't know when the physician wants the patient to go home. The PFN helps with that and gets the prescriptions sent the night before.”– Program Leadership*

“*The relationships between the (program) coordinator and the PFN [are] critical.”– Program Supervisor*

Likewise, many interviewees who did not work directly for the program cited the positive attitudes of the pharmacy program staff as an important facilitator to successful implementation.

“*The enthusiasm from staff stuck out to me. I'm sure they had a lot of challenges on their end but my impression was that it was a lot of work but I never got the impression they didn't have time for me or my patient. There was always someone to answer the phone. It never rang and rang and rang. They never appeared overwhelmed. It would have been a big barrier if there was a ‘you're in the queue’ attitude or a ‘we're just trying to get through the day.’ But they always had a positive attitude.”– Attending*

“*I also liked that they were pretty available from the get-go. They started with very reasonable hours. It was impressive. They weren't just available during business hours. I just liked that it was comprehensive to start. It was a complete package. A lot of pilot programs start small and build up and it takes a long time. (The Program) bit off a big chunk and they delivered. It was also great the speed at which they expanded. It was rapid. They expanded very quickly.”– Attending*

“*The two (program) pharmacists at the beginning were extremely helpful. We knew that they would do what was right for the patients and they could trust them them…The pharmacists were super responsive, helpful, and would work hard for our patients.”– Attending*

“*The (program) pharmacy was really good about listening to each PFN and listening to what works and what doesn't work and adapting.”– Patient Flow Nurse*

### Maintenance

Each stakeholder was asked if they believed the program was sustainable and what needed to happen to bolster its sustainability. Numerous stakeholders cited the need to continually educate hospital providers about the program in order for it to be sustained.

“*I think you always have to change to sustain. You can't take your finger off the pulse. You always have to keep reselling and re-educating and keep the communication lines open. I think they've done a good job in the pharmacy doing this but it has to continue—getting input from stakeholders, listen to them, and give feedback.”*– Nursing Administration.

“*We can't have sustainability without progress. We must continue to actively market the program, keep constant communication and education going. Still need to ‘make a presence’ though. (The Hospital) has high turnover, residents come through, and suddenly no one knows about us.”*– Program pharmacist.

“*The education is on autopilot for people who are already using the program but for new residents, thorough education needs to take place.”* – Patient Flow Nurse.

In addition to the need for recurring education and outreach, stakeholders cited the need for more resources such as staff and physical space to support the program long-term.

“*With more volume, more demand for staff, new staff means training, training is hard in a busy pharmacy location so training sometimes fall to wayside, which can cause staff issues. It's helpful that we hired pharmacist manager and tech coordinator to oversee staff. I don't know if we needed them in the beginning. They may not have been necessary. It was when more staff were hired that the need for oversight was needed.” – Program Pharmacist*

“*Training takes a lot of time, too. We've had major staffing changes. We have to be prepared for those changes, though, and make a comprehensive training plan. But even that means having staff available to do that.” – Program Leadership*

“*The physical space of the pharmacy is important. It's very challenging to get a physical workflow in order. “Everything has to be like McDonald's. Everything moves in a certain order.” We have that now but if they change our space that will be a problem. We're constantly trying to improve things and find ways to make things better. We have no bureaucratic red tape to go through in terms of changing our physical space. We have good autonomy. But to hire more staff and get more space, we need approval. It's not sustainable if administration doesn't listen to the issues we're bringing up. When your team is asking for certain things…”– Program Pharmacist*

“*We have to find space dedicated to Meds to Beds. We have reworked, and reworked, and triple worked our space over time to make it more streamlined and efficient.” – Program Leadership*

### Other

The results thus far represent common themes across the interviews and key points are summarized in Table 1. However, some stakeholders made points that, while not made by others, we believe are important and unique contributions to understanding the implementation of hospital-wide programs. These comments can be grouped into two categories: (1) unanticipated structural or policy challenges and (2) advice for others implementing programs in the hospital setting.

**Table 1:** Summary of key findings by RE-AIM construct.

**Construct**	**Finding**	**Quotes**
Reach	The opt-out model was a facilitator to patients using the program.	“*The opt-out model is extremely important. The patient is sick, the caregiver is in the room tapping their foot, and everyone just wants to go home.” – Pharmacy Administrator*
Effectiveness	Not assessed	
Adoption	Stakeholders have different and specifically competing priorities.	“*For physicians it is all about readmissions and why readmissions occur–patients not getting prescriptions filled, reasons why they don't get prescriptions filled. And how we help with those things.” – Program Pharmacy Supervisor* “*[I tell the nurses] it will help decrease your work[load] and make your discharges quicker.” – Patient Flow Nurse 1, describing how she sells the program to other nurses*
Implementation	Structure of hospital beds/units/providers made educating providers about program difficult.	“*Every unit has a different process for discharge. We went live on 7 North [service] because they had some familiarity with the Meds to Beds process then we went to the Riven [Hospitalist] Services. But Riven is everywhere with certain beds on certain units. So we had to pull back and rethink.” – Program Leader* “*So we decided to roll out to everywhere and then go around and educate everyone. Because not being live on all floors was causing problems.” – Program Leadership*
Maintenance	Education of providers also necessary for sustainment	“*The education is on autopilot for people who are already using the program but for new residents, thorough education needs to take place.”* – Patient Flow Nurse “*We can't have sustainability without progress. We must continue to actively market the program, keep constant communication and education going. Still need to ‘make a presence’ though. (The Hospital) has high turnover, residents come through, and suddenly no one knows about us.”* – Program pharmacist

#### Unanticipated Structural or Policy Challenges

Pharmacy operational leadership noted that when adaptations needed to be made to the program, even though they were in a position to authorize such adaptations, the implementation of those adaptations was dependent upon others. In reference to a decision to start using the tablet computers to deliver the education to patients rather than in-person:

“*We want to do education. We have to do it. But we have people running all over the hospital. So [program leadership] asked, ‘Why can't we use an iPad?’ I thought it was a great idea. What took a long time, though, was getting the device compliant with HIPAA and PHI. We approved the idea quickly and then it took a long time to actually implement it.”*– *Pharmacy Administrator*

In reference to a request from program leadership for more physical office space:

“*[Program leadership] brought me the idea about how they were running out of space but could remodel the store room in the pharmacy relatively easily. And that was a quick decision because I just said ‘Go do it’.” – Pharmacy Administrator*

Certain classes of medications also presented unique challenges that were unanticipated.

“*The controlled substances were an issue before [the new EMR] because they required a paper prescription and there were issues with printing, because the printers were tied to the units but I may be printing a prescription on one unit for a patient on another unit and then I have to get that paper prescription from the other unit, sign it, then find a tube [the pneumatic tube system that physically sends a paper prescription that had to be hand-signed to the pharmacy].”– Attending*

“*Patients who get prescriptions [from the hospital] for narcotics must fill those prescriptions in Tennessee so for out-of-state patients, they can't get it filled at home. So [the program] helps get them filled in Tennessee.”– Patient Flow Nurse*

“*There have been issues with narcotics and blood thinners in the past so the medications were being delivered to the nursing station but that caused downstream problems because the patients have to be notified that their meds have arrived and have to be educated and some patients were then getting upset that they weren't allowed to leave or have their medications. In other instances the patients were going to jail after discharge and telling them where their meds were was causing problems because the [police] officers didn't want them to know they were going to be discharged and then going to jail.”– Unit-Based Clinical Pharmacist*

#### Advice for Others Implementing Hospital-Wide Programs

Every interviewee was asked what advice they had for others who are planning to implement a program hospital-wide.

“*I think they have to get stakeholders early so they understand their perspectives. They need to know who the stakeholders are even. The pharmacy didn't know who to get, who to bring in. So finding someone who can match up the right stakeholders with the project, know who does what roles and who they should talk to. Because you have to have the ability to pitch to key institutional stakeholders. It makes a huge difference.”– Attending*

“*In future roll-outs, just rip the band-aid off and realize that you're going to experience some kickback but have enough staff—too many staff is not a bad thing—to handle that*. ” –* Unit-Based Clinical Pharmacist*

“*You have to set clear expectations with nurses and physicians. Because if they think something is going to take 30 minutes and you don't tell them otherwise then when it takes longer you look like you failed.” – Program Supervisor*

“*In the future, if we're rolling out a new initiative my advice would be to get a champion in administration, sell them on it, then have that trickle down effect. It has to be a top-down approach. Sure, there will be growing pains but it's going to take a lot of selling and talking to the same people over and over again. We need to be a permanent fixture on the floors.” – Program Pharmacy Supervisor*

## Discussion

We found considerable interaction between the different levels of implementation barriers and facilitators, consistent with the findings of Geerligs and colleagues (5). The tension between physicians and nurses on what they prioritized about the program demonstrates this interaction. The program appealed to physicians because they believed it provided their patients with important services such as education about their medications and logistical or financial assistance in getting their medications. This staff-level desire to provide quality care interacted with the system-level desire to reduce readmissions. In this instance, this interaction was beneficial. The staff-level facilitator was in agreement with the system-level facilitator—providing quality care can reduce readmissions (7). However, our results also demonstrated when these interactions can be in conflict. The program appealed to nurses because they believed it would help them discharge patients faster (because the program would provide the medication education to the patient). This staff-level facilitator is in conflict with the system-level desire to reduce readmissions because discharging patients too quickly is associated with readmissions (8).

Another example of the interaction between system-, staff-, and intervention-level barriers and facilitators is the challenge of rolling out the program hospital-wide. The hospital structure and staff fluctuations represented structural barriers to implementation which conflicted with the intervention-level barrier of needing to provide detailed education to providers so they understood the program. The intervention required that program staff provide thorough instructions and education to providers who would be using the program, but the structure of the hospital nursing units and medical services made this difficult. This need to provide detailed education on a continual basis for implementation and sustainability purposes is especially pertinent within the context of continuing medical education (CME). In its most recent strategic plan, the Accreditation Council for Continuing Medical Education noted its plan to evolve CME to include not just clinicians but other members of the healthcare team as well the healthcare institution as a whole (9). Given the interdisciplinary nature of an intervention such as the one discussed here, there is likely an opportunity to incentivize or bolster education around the program by coupling it with CME. This could also improve the tracking and evaluation of implementation outcomes because participation would be better recorded.

This study is also significant because of the inclusion of sustainability data, something Geerling and colleagues noted many hospital-based implementation studies did not examine. At the time of data collection, the program had been operating hospital-wide for more than 2 years, which provided a unique opportunity to study the sustainability of a program in a hospital, where a version of a program may generally be more short-lived due to the use of rapid-cycle quality improvement methods (e.g., Plan-Do-Study-Act cycles). We collected sustainability data with the Maintenance construct of the RE-AIM framework. In the interviews we found that the points interviewees believed were most critical to the sustainability of the program were to address or mitigate the cited implementation barriers and continue or bolster the implementation facilitators. For example, the timing of medication delivery was the most commonly cited barrier to implementation and was cited as an ongoing issue. When asked about what the program needs to do in order to be sustained, not surprisingly many interviewees said the timing issue needed to be resolved. Likewise, when asked what the program “had going for it” in terms of sustainability many interviewees said the positive attitudes of the program staff and the effective communication between providers and the program staff. These were also cited as facilitators to implementation.

This relationship between barriers and facilitators and sustainability has multiple implications. First, it suggests that how programs address barriers is important not just for implementation but also sustainability, which is consistent with other findings (10). This also suggests that, despite interviewees reporting that if the program fails once some providers will not give it a second try, providers will indeed continue to use the program even if a commonly cited barrier remains. This may be because the facilitators to implementation—the positive attitudes of program staff and the effective communication between providers and the program—was also ongoing and thus balanced out the ongoing barrier. However, because the timing barrier was cited as a potential impediment to sustainability, it is unclear how long this “grace period” for barriers will continue.

The last important finding we will discuss is how counterintuitive the program's rollout was. Existing guidance found in the quality improvement and implementation science literature suggests that programs should be rolled out in phases, starting with small-scale change working up to system-wide rollout (11). While this program attempted to do this, the leadership quickly learned that the sequential rollout was causing so much confusion and difficulty that they had to go straight to system-wide rollout. The system-wide rollout presented different challenges because there was suddenly an overwhelming demand for the program, which put strain on program staff. These results suggest that others who are planning to implement hospital-based programs may benefit from considering the pros and cons to a sequential vs. system-wide rollout ahead of time rather than assuming a sequential rollout is the most appropriate plan.

This study is not without limitations. First, this was part of a small-scale evaluation project which placed certain restrictions on the data collection procedures including what data were collected, how they were collected, and the number of interviews conducted. However, we believe our close relationship with the two program leaders in identifying key stakeholders to interview and our rigorous data analysis process help to mitigate this limitation. Second, given the important adaptation to the program that had to be made involving pharmacy technicians, it is a limitation that a pharmacy technician was not interviewed. Because we were not aware of this important adaptation until the data analysis phase, we could not go back to collect more data from this important stakeholder group.

## Conclusion

This study begins to fill two gaps in the implementation literature: it includes assessment of sustainability in hospital settings and sheds light on the implementation of pharmacy-led, bedside medication delivery interventions which are growing in popularity. Results indicate that there are unique challenges both to implementation in a hospital setting and that barriers and facilitators present during early implementation phases may not be resolved and yet the program can still continue for an extended period of time. However, it is unknown how long the program can sustain with unaddressed barriers and facilitators. More work is needed to better understand the relationship between implementation and sustainability and the results of this study can serve as guidance for this future work.

## Data Availability Statement

The datasets generated and analyzed during the current study are not publicly available due concerns about confidentiality with the limited number of participants but are available from the corresponding author on reasonable request.

## Ethics Statement

This project was reviewed by the Institutional Review Board at Vanderbilt University Medical Center (called the Human Research Protections Program) and was determined not to be human subjects research (it was deemed a quality improvement project instead) thus informed consent was not required.

## Author Contributions

BP and SK conceptualized the study. BP collected all data. BP, AM, and SK analyzed the data, interpreted the results, and contributed to the writing of the manuscript. All authors read and approved the final manuscript.

### Conflict of Interest

The authors declare that the research was conducted in the absence of any commercial or financial relationships that could be construed as a potential conflict of interest.
