# Supplementary material for: Implementation and Sustainability of a Pharmacy-Led, Hospital-Wide Bedside Medication Delivery Program: A Qualitative Process Evaluation Using RE-AIM
Source: Front Public Health. 2020 Jan 22;7:419. doi: 10.3389/fpubh.2019.00419 (PMC6988304; doi:10.3389/fpubh.2019.00419)
Supplement: Supplementary file 1 [file Data_Sheet_1.PDF]

Meds to Beds Interview Codebook

1. *Reach:* Code for this if someone is discussing the number of patients receiving Meds to Beds. Do not code for this if the discussion is about providers using the program. Reach is specific to patients receiving Meds to Beds, not providers using the program. Reach is defined by RE-AIM as "the extent to which the program is received by its target group".

2. *Effectiveness:* Code for this if someone is discussing how effective they thought the program was at any outcome (i.e., patients' are guaranteed to leave hospital with their medications, increases patients' ability to adhere to medications, helps patients pay for medications, patients receive medication counseling, etc.). This must be concrete effectiveness such as "My patients are leaving with their medications more than they used to." Do not code for this if someone is describing what they *perceive* as the positive outcomes of the program. They must be describing outcomes they have witnessed.
    a. *Effectiveness related to scale-up:* Code for this if someone is discussing how effectiveness relates to the scale-up of the program from one unit to another or throughout the entire hospital.

3. *Adoption:* Code for this if someone is discussing providers use of Meds to Beds. This includes what was done to "sell" the program to providers and providers' decisions to use or not use the program. Do not use this code for barriers and facilitators (see Code 4 Implementation).

4. *Implementation:* Code for this if someone is discussing the barriers, challenges, facilitators, enablers to the implementation of Meds to Beds (i.e., what went wrong, what went right). This can include barriers/facilitators providers experienced or that was observed as patients experiencing. Code for this if someone is discussing the adaptations or changes that were made to the program since it began. Do not use this code for things someone thinks need to change in the future with the program (see Code 6 Maintenance).
    a. *Implementation related to scale-up:* Code for this if someone is discussing how implementation relates to the scale-up of the program from one unit to another or throughout the entire hospital.

    b. *Barriers to implementation:* Describe the nature of any barrier (e.g., unclear who to contact, competing demands, timing, etc).

    c. *Facilitators to implementation:* Describe the nature of any facilitator (e.g., good communication, transparent, positive attitude, etc.)

5. *Maintenance:* Code for this if someone is discussing what they think the program has going for it looking forward, or what they think needs to change or improve going forward. This is different than barriers and facilitators that were experienced in the past. Use this code specifically for barriers and facilitators that are anticipated and are specific to the maintenance or sustainability of the program.
    a. *Maintenance related to scale-up:* Code for this if someone is discussing how maintenance relates to the scale-up of the program from one unit to another or throughout the entire hospital.
