# Supplementary material for: Implementation and Sustainability of a Pharmacy-Led, Hospital-Wide Bedside Medication Delivery Program: A Qualitative Process Evaluation Using RE-AIM
Source: Front Public Health. 2020 Jan 22;7:419. doi: 10.3389/fpubh.2019.00419 (PMC6988304; doi:10.3389/fpubh.2019.00419)
Supplement: Supplementary file 2 [file Data_Sheet_2.PDF]

<u>INTERVIEW</u>
Date:
Name:
Title:

- **What is your role with the program?**

- **Have you been involved since the beginning? How did you become involved?**

- **How do you describe the program to others? Are there different ways you "pitch" it depending on the person?**

- **What were some barriers to implementing the program?**
  Probe: What were the barriers at the beginning? How did they change as more providers on different units started using the program?

- **What were the facilitators to implementing the program?**
  Probe: What were the facilitators at the beginning? How did they change as more providers on different units started using the program?

- **What adaptations had to be made to successfully implement the program? How did you decide when and how to make these adaptations?**

- **How much has the program changed from its original form one year ago?**

- **Do you think the program is sustainable in its current form?**

- **What do you think needs to happen to make it more sustainable?**
